# Nipah Virus Outbreaks in Kerala: An Impending Doom?

**DOI:** 10.1002/hsr2.70195

**Published:** 2024-11-11

**Authors:** Vivek Sanker, Faheem Vellekkat, Tirth Dave

**Affiliations:** ^1^ Department of Neurosurgery Trivandrum Medical College Kerala India; ^2^ Indira Gandhi Medical College and Research Institute Puducherry India; ^3^ Bukovinian State Medical University Chernivtsi Ukraine

**Keywords:** bats, India, Kerala, Nipah virus, zoonotic disease

## Abstract

**Background and Aims:**

The resurgence of Nipah virus (NiV) in Kerala, India, represents a significant public health challenge. This paper aims to explore ongoing challenges associated with NiV transmission, focusing on environmental factors, healthcare responses, and shifts in clinical manifestations. We also highlight the critical need for proactive management strategies to prevent future outbreaks, given the virus's zoonotic nature and evolving transmission dynamics.

**Methods:**

A search was conducted using keywords such as “Nipah virus,” “Kerala,” and “bats” in PubMed, Scopus, Google Scholar, and trusted news sources. Articles and reports were selected based on their relevance to NiV transmission, clinical presentation, and containment efforts, particularly concerning recent outbreaks in Kerala.

**Results:**

Recent outbreaks have shown atypical respiratory presentations, complicating early detection and increasing the importance of healthcare containment. The virus's clustering within specific regions and the emergence of a new genotype underscore the need for enhanced surveillance.

**Conclusion:**

The frequent NIV outbreaks in Kerala demand a multifaceted approach to prevention. Early detection systems, public awareness campaigns, and rigorous environmental management are essential. Collaborative efforts between government and public health entities are paramount to mitigate the impact of this deadly virus. Continuous research is imperative to safeguard public health.

Dear Editor,

Since May 2018, there have been four outbreaks of the Nipah virus (NiV) in Kerala, India, with the most recent one being in late August 2023 (Table 1). Three of these outbreaks—out of four—have been contained to the Kozhikode district. Although NiV has been found in a variety of fruit bats (*Pteropus medius*) across several districts in Kerala [1], the causes of this surge and clustering of *NiV* outbreaks are still unclear and require further evaluation.

**Table 1:** Summary of the various outbreaks of Nipah virus (NiV) in the state of Kerala.

Sl. no.	Outbreak	Date reported/duration	Location	No. of cases	Deaths
1	First outbreak	May 19, 2018–June 10, 2018	Kozhikode District	23	21
2	Second outbreak	June 4, 2019	Ernakulam District	1	0
3	Third outbreak	September 5, 2021	Kozhikode District	1	1
4	Fourth outbreak	August 2023– September 15, 2023	Kozhikode District	6	2

The Ministry of Health and Family Welfare, Government of India, reported six cases of NiV infection, resulting in two fatalities, within the timeframe of September 12–15, 2023, in Kozhikode district, Kerala (Figure 1). The primary case had an unidentified source of infection, while subsequent cases were either familial or associated with the initial case within a healthcare setting. As of September 27, 2023, a total of 1288 contacts linked to the confirmed cases underwent identification and quarantine for 21 days, encompassing high‐risk contacts and healthcare personnel. Among the 387 samples analyzed since September 12, only six tested positive for NiV infection, with no additional cases reported post September 15. The Government implemented containment measures in nine villages of Kozhikode district, including movement restrictions, social distancing measures, and mandatory mask usage in public spaces. Furthermore, major public events in Kozhikode district remained prohibited until October 1, 2023, and neighboring districts and states were advised to enhance surveillance, as communicated by the National Institute of Virology, Pune [2].

**Figure 1:**
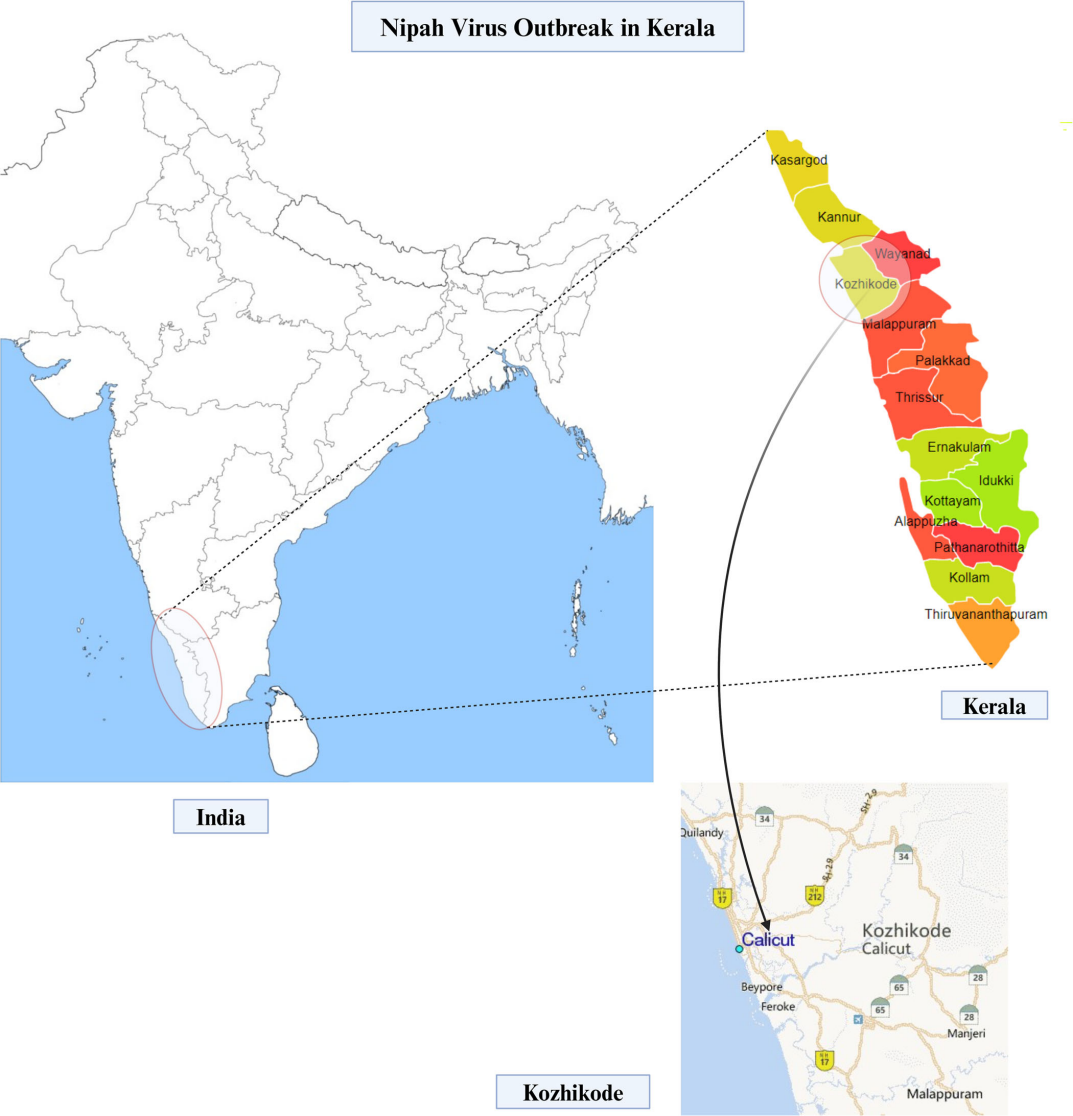
Location of Kozhikode district in Kerala, India, where the 2023 Nipah outbreak occurred (created with BioRender).

NiV infection is a zoonotic disease that can be transmitted to humans through contact with animals that carry the virus, such as bats and pigs. Human‐to‐human transmission is also possible, but less frequent. The infection can cause severe clinical manifestations, such as acute respiratory infection and fatal encephalitis (Figure 2). The main strategy to reduce or prevent human infection is to increase awareness of the risk factors and preventive measures among the population. The treatment of patients should focus on providing supportive care and intensive support for severe respiratory and neurologic complications (Figure 3).

**Figure 2:**
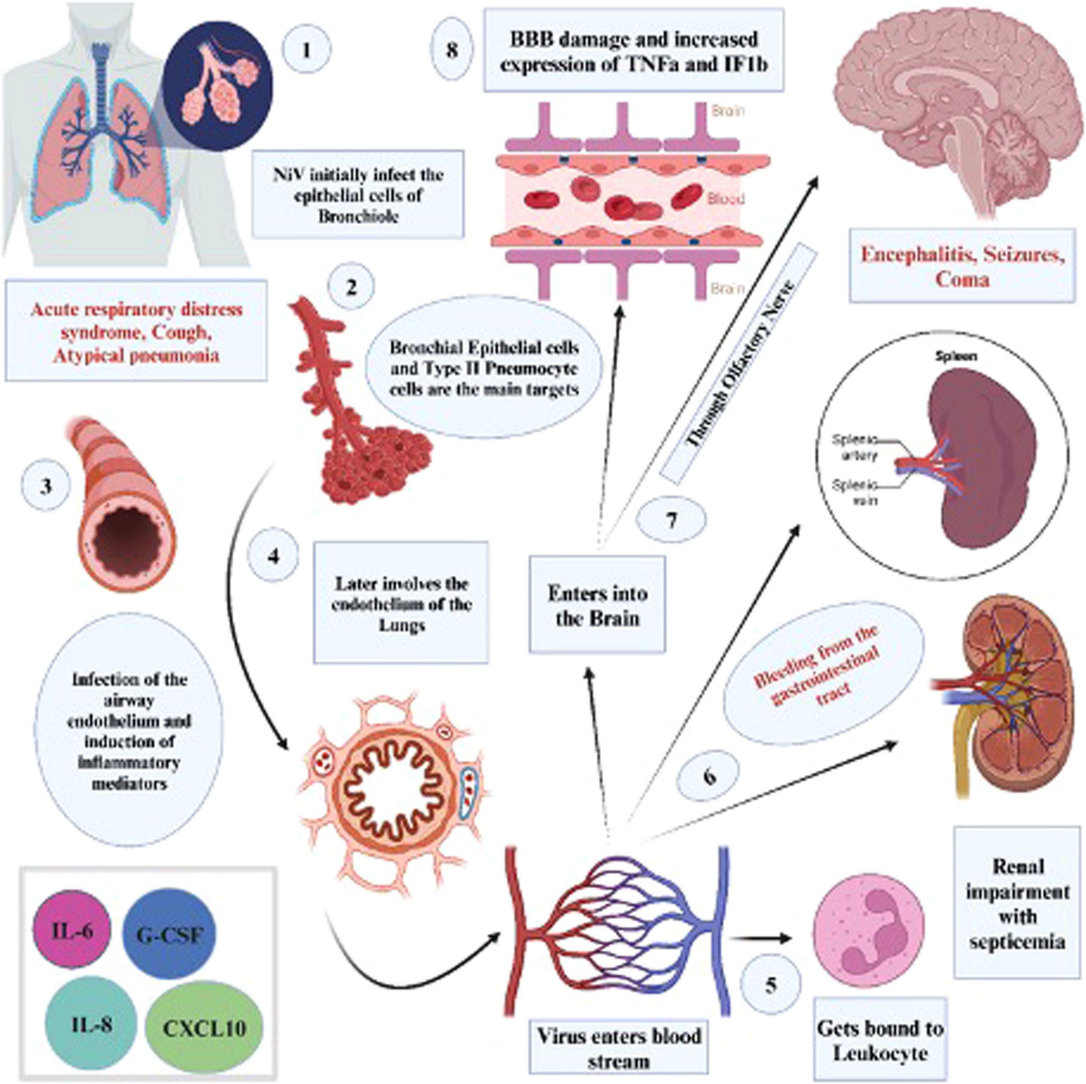
Intricate pathogenesis of Nipah virus (NiV) infection in humans. It provides a detailed overview of how NiV affects various human organs and systems, and the associated clinical manifestations (created with BioRender).

**Figure 3:**
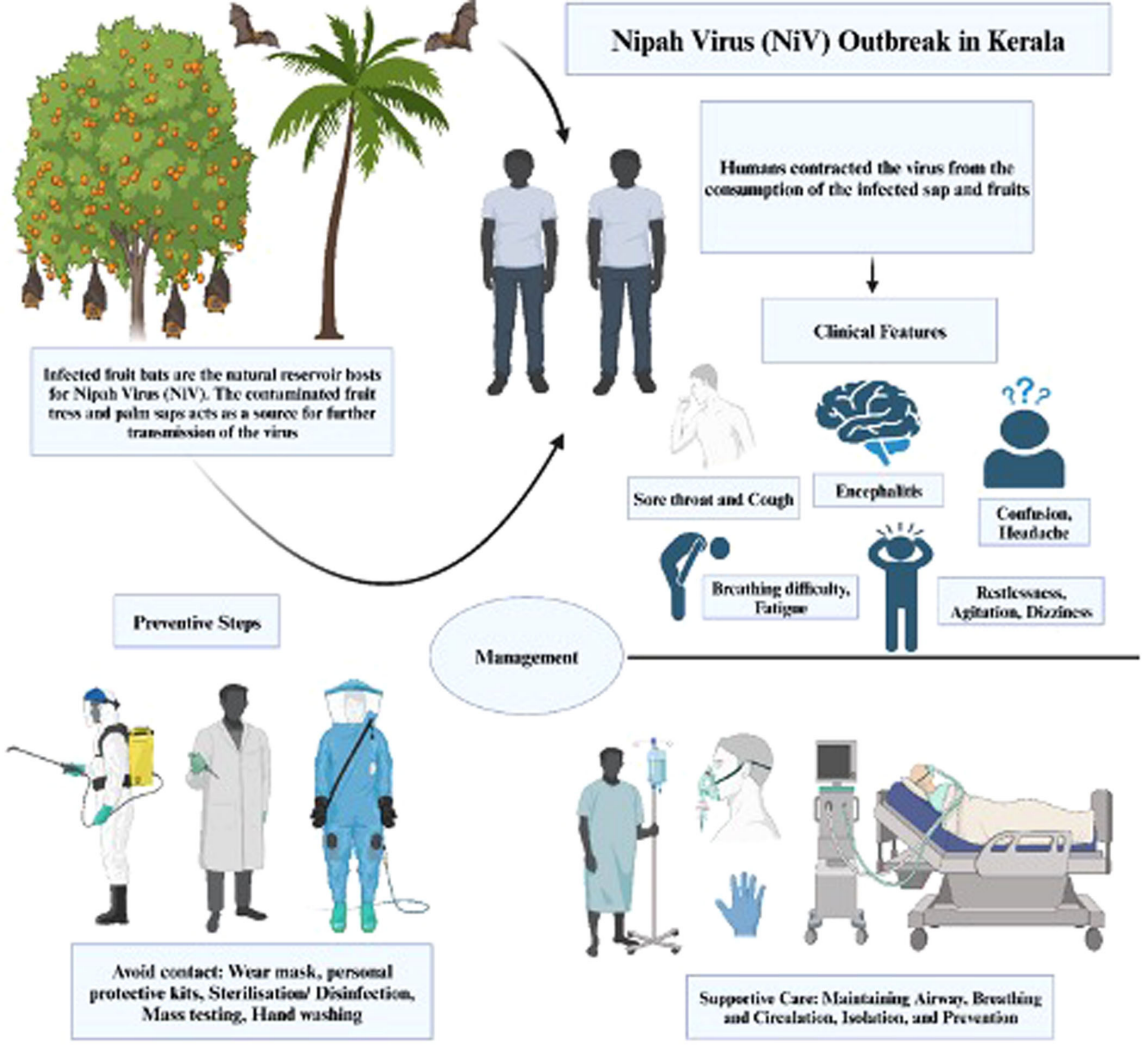
A comprehensive visual summary of the NiV outbreak in Kerala, focusing on clinical features, management, and preventive strategies (created with BioRender).

It is the sixth NiV outbreak in India since 2001. Recurrent outbreaks of NiV have occurred in several Southeast Asian countries, including Malaysia, Bangladesh, and India. In Bangladesh, consumption of date palm sap contaminated by bat saliva or urine was found to have caused NiV outbreaks. In Malaysia, pigs acted as intermediate hosts and transmitted the virus to humans through direct contact. However, in Kerala, the route of spillover from bats to humans remains unclear. The outbreaks in Kerala were also highly lethal, with a case fatality rate of 90%, compared to 40% in Malaysia and 70% in Bangladesh. During the 2018 NiV outbreak in Kerala, which was localized in Kozhikode and Malappuram districts, 17 lives were lost. Another outbreak in 2021, limited to the village of Pazhur in the Chathamangalam gram panchayat of Kozhikode district, resulted in a single fatality on September 5, 2021 [3] https://english.mathrubhumi.com/amp/news/kerala/nipah-no-indication-of-second-wave-yet-says-kerala-health-minister-1.890922 . A new genotype that is independently emerging in Southern India has been detected by phylogenetic analysis of the NiV nucleocapsid (N) gene sequences from Kerala, which show that they belong to a different cluster than those from Malaysia and Bangladesh [4].

NiV antibodies in bats have been detected from several states in India [5], but only Kerala has experienced four successive human NiV outbreaks. This finding was also revealed by a nationwide survey conducted by the National Institute of Virology of the Indian Council of Medical Research (ICMR) in Pune, which tested bats across nine states and one Union Territory for NiV antibodies [6]. The survey found that bats from Kerala, Tamil Nadu, Karnataka, Goa, Maharashtra, Bihar, West Bengal, Assam, and Meghalaya, as well as the Union Territory of Pondicherry, were positive for NiV IgG antibodies. IgG antibodies may aid in providing proof of historical circulation in bat populations. However, the absence of conclusive antigen detection and IgM antibodies suggests that the virus may not be actively spreading in ways that lead to human outbreaks.

There are two potential explanations for the rise in NIV outbreaks in Kerala during the past 5 years: either an increased effective spillover of the virus from bats to humans or the state's healthcare system's efficient screening and reporting of cases. However, since fruit bats with *NIV* antibodies have been found in other States as well, it is likely that NIV infection and deaths are occurring elsewhere in India but remain unnoticed, while they are identified and contained in Kerala, especially in Kozhikode district. Unlike the typical encephalitic presentation of Nipah infection, the recent outbreak showed a novel manifestation of pure respiratory symptoms, which has not been reported anywhere else in the world. This could only be identified due to a high index of suspicion.

Pragya Yadav, who leads the Nipah research team at India's National Institute of Virology, attributed the enhanced viral transmission from bats to humans to the loss of natural habitats, decreased biodiversity, and animal movement brought on by population growth. One of the hotspots for these spillover episodes is Kerala, a coastal state with over 40 bat species and 35 million residents. Its hilly forests and woodlands, where bats thrive, have been gradually cleared for various development projects, including transportation and industrialization. In comparison to 58% in 2002, 83% of Kerala was vulnerable to spillover at the time of the 2018 Nipah outbreak, according to a Reuters investigation [7]. The high fatality rate and the frequent outbreaks—four outbreaks in 5 years—underline the pressing need for rigorous research and analysis of the fundamental causes of the recent NiV outbreak in Kerala.

An international team of scientists used data from 2000 to 2018 to identify Kerala as one of the top seven “global hotspots” for the potential emergence of a novel coronavirus related to SARS [8]. They attributed the spillover of zoonotic infectious diseases to major factors such as deforestation, high livestock density, and human intrusion into bat habitat. However, NIV was the first to emerge in this region. Malik Fasil Madala, a wildlife ecologist who research flying foxes at Kerala Agricultural University, witnessed the degradation of the bats' habitat due to mining and construction activities. He referred to studies that showed that stress from such disturbances impairs the bats' immune system and makes them more vulnerable to viral infections [9].

The high mortality rate and the frequent occurrence of four Nipah outbreaks in 5 years highlight the urgency of conducting rigorous research and analysis on the potential causes of the recent NiV outbreak in Kerala. A crucial initial step is to establish early detection systems in all countries that are potential hosts of NIV reservoirs. This requires a better understanding of the risk factors and distribution of the virus. The strain that caused the outbreaks in Kerala originated from Bangladesh in 2001, where it was overlooked by health systems due to its high fatality rate and small‐scale or sporadic occurrence. However, the outbreaks in Bangladesh in 2001 and 2003 were only retrospectively confirmed by testing stored samples for Nipah antibodies.

The risk of NIV infection is exacerbated by deforestation and human encroachment into bats’ habitats. Deforestation disrupts ecosystems, pushing bats closer to human settlements and increasing the likelihood of viral spillover. Human intrusion into these habitats further amplifies the risk as it brings people into closer contact with potentially infected bats, contributing to the transmission of the virus. Addressing these environmental factors is crucial in mitigating the threat of NIV outbreaks and preserving the delicate balance between human activities and wildlife habitats [10] (https://www.nationalgeographic.com/science/article/deforestation-leading-to-more-infectious-diseases-in-humans).

To prevent future NIV outbreaks in India, collaborative efforts between the government and the public are essential. Government measures include controlling land development and safeguarding animal habitats in high‐risk areas, monitoring bats closely, protecting their habitats, enhancing surveillance of animals and people in NIV‐prone regions, and prioritizing accelerated research and development for the virus. On an individual level, practicing regular hand hygiene, avoiding contact with sick bats or pigs, refraining from consuming products potentially contaminated by bats, such as raw date palm sap or fallen fruit, and promoting awareness of risk factors and preventive measures are crucial steps to mitigate the risk of NIV transmission [11, 12, 13, 14, 15] (https://www.reuters.com/investigates/special-report/global-pandemic-bats-prevention/, https://www.cdc.gov/vhf/nipah/prevention/index.html, https://www.who.int/emergencies/disease-outbreak-news/item/2023-DON490, https://www.who.int/news-room/fact-sheets/detail/nipah-virus).

## Author Contributions

**Vivek Sanker:** conceptualization, writing–review and editing, writing–original draft, supervision. **Faheem Vellekkat:** writing–original draft, writing–review and editing. **Tirth Dave:** writing–original draft, writing–review & editing, software; resources.

## Ethics Statement

The author has nothing to report.

## Conflicts of Interest

The authors declare no conflicts of interest.

## Transparency Statement

The lead author Tirth Dave affirms that this manuscript is an honest, accurate, and transparent account of the study being reported; that no important aspects of the study have been omitted; and that any discrepancies from the study as planned (and, if relevant, registered) have been explained.

## Data Availability

The data that support the findings of this article are available from the corresponding author upon reasonable request.
